# The perspectives of healthcare professionals in mental health settings on stigma and recovery - A qualitative inquiry

**DOI:** 10.1186/s12913-022-08248-z

**Published:** 2022-07-09

**Authors:** Savita Gunasekaran, Gregory Tee Hng Tan, Shazana Shahwan, Chong Min Janrius Goh, Wei Jie Ong, Mythily Subramaniam

**Affiliations:** Research Division, Institute of Mental Health, 10 Buangkok View, 539747 Singapore, Singapore

## Abstract

**Background:**

Mental health stigma is one of the most prominent barriers to recovery, and it is widely known that stigma may manifest differentially in different cultures. Healthcare professionals working closely with persons with mental illnesses (PMI) may provide important insights towards stigma that are otherwise unattainable from caregivers and consumers. However, there is a dearth of literature on healthcare professionals’ perspectives on this topic. Thus, this study uses a multilevel approach to explore how stigma affects recovery from the perspectives of healthcare professionals that work closely with PMI in Singapore.

**Methods:**

Semi-structured interviews were conducted with a total of 17 healthcare professionals who were working in mental health settings in Singapore. Participants were recruited via direct email invitation or through snowball sampling. Data collected was analysed with the inductive thematic analysis method. All coding and inter-rater analyses were performed with NVivo.

**Results:**

The current study themes identified stigma-related factors that influence PMI’s recovery from the perspectives of healthcare professionals working closely with PMI. These factors were organised into three overarching themes in a multilevel structure. The three themes were classified as Micro Factors (e.g., internalised stigma), Meso Factors (e.g., discrimination of people associated with the stigmatised group), and Macro Factors (e.g., structural stigma and stigma within healthcare settings).

**Conclusions:**

The findings of this study gave us a greater understanding of how stigma influences recovery in Singapore, which could be used to guide the development and implementation of future policies and strategies to promote recovery. Importantly, our results suggest that improving mental health literacy, addressing cultural misgivings towards mental illness, implementing recovery-oriented practices, and making insurance more accessible for PMI could mitigate the deleterious impact that stigma has on recovery.

**Supplementary Information:**

The online version contains supplementary material available at 10.1186/s12913-022-08248-z.

## Introduction

The term recovery has been gaining traction in the mental health field in recent years, and has also become the guiding principle for mental health systems in many countries [1, 2]. Recovery has been largely defined as “a deeply personal, unique process of changing one’s attitudes, values, feelings, goals, skills, and/or roles” and “a way of living a satisfying, hopeful, and contributing life even within the limitations caused by illness” [3, 4]. In recent years, recovery-oriented practices have shifted towards being more individual-focused and centred around helping people lead meaningful lives [4]. However, there has yet to be a consensus on what the term means, as evinced by a review that suggested that there are differing views among persons with mental illnesses (PMI), caregivers, and service providers on what recovery entails [5]. Likewise, the literature suggests that there are also differing opinions on the factors that impact the recovery of PMI [6–8]. One of the most widely studied barriers to recovery is the stigma towards mental illness. Angermeyer and Schomerus [9] argued that it is essential to understand stigma concurrently when investigating recovery, as stigma might account for some of the blind spots of recovery, for “where recovery sees challenges, stigma identifies obstacles”. There is compelling evidence in the literature evincing that stigma often affects the recovery of the service users [10, 11]. For instance, stigma may discourage an individual from seeking help due to the fear of being labelled with a mental illness diagnosis [12]. Even amongst individuals who have sought treatment, their recovery may also be compromised by self-stigma [10, 13]. The pessimistic views that healthcare professionals hold towards recovery, also known as therapeutic pessimism, is another form of stigma experienced by those seeking help, which has been demonstrated to exert a pernicious effect on the consumer’s recovery [11]. In mental health settings, therapeutic pessimism is defined as the inclination to believe that PMI are difficult to treat or immune to treatment [14]. Perhaps more troubling is that research has established an association between pessimistic views of recovery and a sense of helplessness for some healthcare professionals, which leads them to believe that ‘‘what they do doesn’t matter’’ [15], adding to the problem of inadequate treatment provision. Additionally, a corollary to stigma is the reduced opportunities available to consumers and greater social exclusion [16]. Therefore, understanding stigma and its underlying reasons are pivotal in ensuring that interventions designed to reduce stigmatisation target specific underlying issues that serve as barriers to PMI’s recovery.

Stigma toward PMI is not the only factor that impacts recovery – stigma experienced by healthcare professionals working closely with PMI can also indirectly contribute as a barrier to PMI’s recovery. In recent years, there is a growing interest in associative stigma experienced by healthcare professionals in mental health settings, whereby these professionals are judged with similar stigmatising stereotypes as their patients [17]. This was largely explored in a qualitative study by Vayshenker and colleagues, postulating that associative stigma experienced by these healthcare professionals can lead to severe consequences in the quality of care provided to PMI [18]. To be more specific, the established link between emotional exhaustion, job dissatisfaction, and associative stigma might lead to diminished empathy towards PMI [18]. For example, the study revealed that factors such as job devaluation (e.g., minimizing the training required for mental health professionals) contributes to feelings of frustration and burnout among these healthcare professionals [3]. Other studies have also revealed that psychiatric nurses were deemed as less skilled and valued or even viewed as not “real” nurses [19–21]. These stereotypes associated with mental health professionals not only devalues the role these individuals play in treatment and recovery but also underplay the needs of PMI in the healthcare system. In addition, these stereotypes can further aggravate stigmatising beliefs about mental health conditions [15]. Other studies have similarly found that stigmatisation by association influences professional burnout, depersonalisation, lower job satisfaction, and emotional exhaustion among healthcare professionals working with PMI [20, 22–24]. PMI also described higher self-stigma and decreased satisfaction in healthcare institutions when their healthcare professionals experience more associative stigma [17]. Extensive literature has also exhibited the link between work stress and performance, indicating that stress in the workplace because of stigmatisation influences interpersonal performances, such as reduced sensitivity toward PMI and increased disregard of individual differences among PMI [20]. Thus, they may be less likely to provide quality services to their clients, serving as a barrier to recovery [18].

Despite the role of healthcare professionals in understanding mental health stigma and its impact on recovery, a look into current literature reveals a pattern of investigating stigma and recovery from the standpoint of service-users and the general public, with a scarcity of research done to address the perspectives of healthcare professionals working in mental health settings [25, 26]. This is surprising in many respects, considering that PMI regularly interact with these healthcare professionals [18]. There are certain advantages to understanding stigma and barriers to recovery through the perspectives of healthcare professionals. Firstly, as compared to consumers’ and caregivers’ perspectives, healthcare professionals may sometimes be able to provide more objective third-party insights, such as in contexts where palpable tensions exist between the consumer and the caregivers or when caregivers are overprotective [27]. Secondly, although PMI are the most important individuals to discuss barriers and facilitators to recovery, they may sometimes also possess poor insight towards their mental illness such as a lack of awareness of their symptoms, significance, and severity of their illness, which may be associated with poorer perceptions of experienced stigma [28–30]. Furthermore, a study by Happell and colleagues reported that consumers felt that their recovery was hindered when healthcare professionals prioritised treating them according to symptoms instead of their individual needs [31]. Hence, understanding recovery and stigma from healthcare professionals’ perspectives may elucidate some insights as to whether it matches consumers’ expectations. Most importantly, healthcare professionals are often present in situations where they can witness significant breakthroughs and outcomes in patients which surpasses expectations [27, 32]. For these reasons, it would be beneficial to consider healthcare professionals’ viewpoints on the barriers and facilitators to recovery.

According to Slade et al. [4], it is important to investigate factors that enable or hinder recovery within a non-Western cultural context to develop more culturally relevant recovery concepts that can better address the needs of service users. To our knowledge, there are limited publications in the literature about the topic of recovery in Singapore [33, 34], a country in Southeast Asia where the lifetime prevalence of mental illness is reported to be approximately 13.9% [35]. Mental health services in Singapore are delivered both in hospitals and at the community level. The Institute of Mental Health (IMH) is the only state-run psychiatric hospital comprising in-patient and out-patient services. Public and private hospitals deliver inpatient and outpatient mental health services also but in small-scale capacities [36]. In the community, mental health services are delivered by primary care physicians in state-run clinics (i.e., polyclinics) or as General Practitioners (GP), psychologists, and counsellors working in either volunteer welfare organisations that provide care to PMI or educational institutions and other settings [36]. A nationwide survey reported considerable stigma towards PMI in Singapore among the general public [35], and stigma has also been surmised to be a contributor to the wide treatment gap in Singapore. Treatment gap is defined as the absolute difference between the prevalence of a particular mental disorder and those who had received treatment for that disorder [37]. Anationwide study revealed that more than three-quarters of individuals (78.6%) did not seek help despite meeting the criteria for a mental disorder [37]. Qualitative evidence in Singapore also indicates that PMI do experience discrimination and prejudice due to stigma [38]. Even though stigma possesses ubiquitous features across contexts, the specific experiences and manifestations of stigma may be localised and vary according to the cultural context [39, 40]. To date, there has yet to be any qualitative study that explored both stigma and recovery in Singapore, specifically from the healthcare professionals’ (HP) perspective. This study aims to utilise a qualitative approach to investigate how stigma affects the recovery of PMI through the lens of HP working in mental health settings in Singapore. Since stigmatising processes operate on multiple levels, the study adopted Logie et al.’s concept of multilevel forms of stigma – micro (intra/interpersonal), meso (social networks/community/norms), and macro (structural/institutional exclusion/discrimination) to understand the determinants of stigma that affect recovery [41].

## Methods

This qualitative study is part of a larger research project that examined the concept of mental illness stigma in Singapore from the perspectives of five stakeholder groups – the general public, PMI, caregivers of PMI, HP in mental health settings, and policymakers [42–44]. As the primary objective of the present study was the provision of actionable knowledge, it therefore adopted a pragmatic approach in health services research and did not assume any specific methodological orientation [45]. The study was approved by the National Healthcare Group Domain Specific Review Board, and written informed consent was obtained from all participants before initiating study-related procedures. For this study, only the data from the healthcare professional’s stakeholder groups were analysed.

### Sample

Participants were recruited from March 2019 to July 2019 through direct email invitations. These individuals were identified through purposive and snowball sampling based on their experience of working with PMI, to represent a range of professions from different organizations. The inclusion criteria for this study comprised (1) being a Singapore citizen or Permanent Resident; (2) being aged 21 years and above; (3) a healthcare professional currently working with persons with mental illness (4) willingness to allow the interview to be audio recorded. Healthcare professionals in our study included professional care providers providing care to PMI in Singapore (e.g., general practitioners, psychiatrists, nurses, and psychologists including those from community-based services and voluntary organisations involved in primary as well as immediate and long-term care). Participants’ duration of work in mental health settings ranged from 5 years to 24 years. Semi-structured interviews (SSI) were conducted with a total of 17 health care professionals in mental health settings. Refer to Table 1 for other sociodemographic information. Data collection ended after 17 SSIs as no new information surfaced regardless of the interviewee’s occupation, indicating that data saturation was reached [46].

**Table 1:** Sociodemographic characteristics

Variable	Mean
Age (in years)	50.1
**Duration of working as HP (in years)**	19.8 (5 to 44 years)
	***N***
0 to 10 years	3
10 years to 20 years	6
21 years to 30 years	5
Above 30 years	3
**Sex**	
Male	9
Female	8
**Ethnicity**	
Chinese	12
Non-Chinese	5
**Highest Education Level**	
University Degree	8
Post-grad Degree	9
**Specific Profession**	
Psychiatrist	4
General Practitioner	1
Psychologist	2
Nurse	1
Counsellor	3
Medical Social Worker	2
Rehabilitation Manager	1
Pharmacist	1
Case Manager	1
Occupational Therapist	1
**Type of setting**	
Public hospitals	12
Educational institutions	2
Volunteer welfare organisations	3

### Data collection

Before the start of the SSI, background information (i.e., age, gender, ethnicity, occupation, number of years working in mental health settings) was collected using a sociodemographic questionnaire. Each SSI was conducted by two study team members – one of whom would be the interviewer and the other a note-taker – and lasted between 1 and 1.5 h. Participants were assured of confidentiality and that there were no right or wrong opinions during the written informed consent process. To ensure that information was captured accurately, the SSIs were audio-recorded and then transcribed verbatim by a member of the study team.

As this study is part of a larger research project, the study team developed a topic guide (see Additional file 1) consisting of open-ended questions which mainly pertained to the stigma of mental illness. The larger research project aimed to explore the concept of stigma from various stakeholder perspectives, to gain a more in-depth understanding of this complex phenomenon in Singapore. Building on existing quantitative research in Singapore relating to stigma, it delved further into specific existing gaps in current knowledge. The study, therefore, sought to identify both specific reasons for stigmatising attitudes, as well as interventions that may help reduce the stigma, which in turn will be used to help inform future anti-stigma initiatives and ultimately reduce stigma towards mental illness. The team developed the questions in the topic guide utilizing the recommendations by Krueger et al. [47] – the recommendations suggested the use of questions that (1) elicit information that is directly associated with the study aims, (2) are neutrally phrased to avoid biases in participants’ responses, and uncomplicated and easy for a layman to understand, (3) can be answered by all participants, (4) do not elicit feelings of discomfort or defensive responses when the participant is answering and (5) are open-ended for detailed responses. Team members conceptualised potential questions pertaining to the objectives of the study, and one study team member drafted the questioning route, reordered, and paraphrased the questionnaire to generate a logical flow. This draft was subsequently circulated among the team members and suggestions were included. Decisions to exclude questions were based on their relevance in eliciting responses to the research objective and all final decisions for the questions were made by the lead investigator (MS). Some of the final items in the topic guide included: “To what extent do you think people can recover from mental illness?” and “Do you think healthcare providers hold negative views towards those with mental illness? Can you tell us more about it?”. All interviewers in the study utilised this topic guide comprising the final questions to ensure a uniform approach to data collection.

### Analysis

The data were analysed using inductive thematic analysis, which includes the familiarization of the data, coding, generating themes, reviewing themes, and defining and naming themes [48]. Preceding the development of the codebook and analysing all data, members of the study team (GTHT, SS, CMJG, WJO, and MS) independently analysed a subset of SSI transcripts and utilised an open coding approach to identify and generate key themes [49]. During this preliminary analysis of SSI transcripts, study team members familiarised themselves with the data and highlighted quotes of relevance in the transcripts which could be grouped into axial codes, before proposing themes representative of an abstract concept which their axial codes could be classed under. Themes for the preliminary codebook were then generated via an iterative process of grouping axial codes with similar concepts into themes based on their common properties, while axial codes with the distinct concept were grouped to form separate themes. Finally, consensus on any disagreements regarding codes and themes was reached through discussions and iterative review, and the preliminary codebook was thereby developed. The codes were specified with the following: label, definition, inclusions and exclusions, and typical and atypical exemplars from the raw data.

Using the preliminary codebook, three members of the study team (GTHT, CMJG, WJO) independently coded three similar transcripts, and held separate meetings after coding of each transcript to discuss their findings (i.e., any codes identified which did not fit into any established themes, or any themes that were found to be redundant). The codebook was updated after each meeting, and the final revised codebook was established after the third coding, when team members agreed themes were useful and accurate representations of the data, no new themes could be derived from the data and had no difficulties coding with the current existing themes. To promote understanding and consistency across the team, the final codebook included definitions and example quotes under each theme. Kappa scores were calculated using the 3 transcripts to measure the level of agreement among the three coders (GTHT, CMJG, and WJO). Upon achieving a satisfactory Kappa score of 0.78 to 0.83 for the third transcript coded [50], the other transcripts were then disseminated to the three coders for independent coding. The results of this study are based on secondary data analysis of the transcripts. Although the main topic explored pertains to stigma, the study team noticed that discussions about the impact of stigma on recovery recurred in all the interviews, which led to the formulation of the research question in this study. The authors (SG and GTHT) then utilised a subset of the coded data (i.e., codes that addressed the impact of stigma on recovery) to develop the themes explored in the results.

Lastly, SG and GTHT organised the final themes into three overarching multilevel categories of stigma – micro (e.g., individual attitudes and beliefs), meso (e.g., community and social norms/networks), and macro (e.g., structural factors including organizational power, laws and policies, health and social service systems) [41]. All analysis and inter-rater reliability tests were performed using Nvivo V.11 and Nvivo V.13. (QSR International. NVivo Computer software).

## Results

**Fig. 1:**
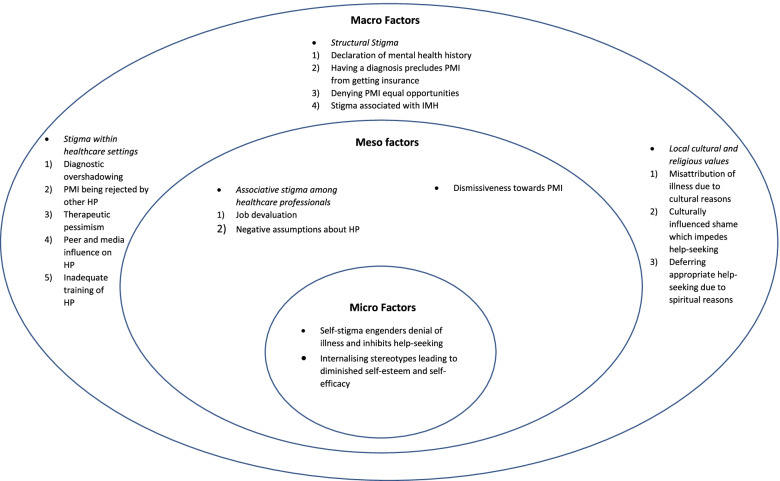
Stigma-related factors that affect recovery organised in a multilevel structure. Semi-structured interviews with healthcare professionals (HP) working closely with persons with mental illnesses (PMI) (*n* = 17) highlighted issues of multilevel (micro, meso, and macro) types of stigma-related factors towards both PMI and HP working closely with them that affect the recovery of PMI

The results of this study were organised into three overarching multilevel categories, which also comprised themes and subthemes (refer to Fig. 1). These themes represent stigma-related factors that influence PMI’s recovery from the perspective of HP. The overarching categories were classified as micro factors (stigma-related factors at the service user’s intrapersonal level), meso factors (how cultural values that exist in the community of the consumers and rejection in social networks impact recovery), and macro factors (stigma in the broader institutional context). To ensure that standard usage of English is maintained, minimally corrected verbatim quotes are presented.

### Micro factors

The results revealed that intrapersonal levels of stigma (micro), which includes personal feelings of shame and internalised stigma, were described by participants – internalised stigma refers to one’s acceptance of negative perceptions and attitudes towards themselves and other PMI [51].

#### Internalised stigma engenders denial of illness and inhibits help-seeking

 Participants provided inputs on how the stigma towards mental illness on a personal level could lead to the denial of illness and even hinder the PMI’s willingness to seek help.

“some don’t even feel that they deserve friends, they feel that they don’t even deserve treatment. Because they say “I have an issue, so what’s the point?” You know? I don’t see…” Maybe you should go and treat those with cancer…” Because mental illness is also invisible? So even if they have it, I think it’s hard to still explain to people that they are just as ill as anybody with cancer, with diabetes, that they deserve the same treatment?” [Psychologist]

#### Internalising negative stereotypes leading to diminished self-esteem and self-efficacy

Additionally, participants also mentioned how PMI may be susceptible to the “why try” effect due to self-stigma [52]. As a result of internalizing negative stereotypes about mental illnesses, PMI’s self-esteem and self-efficacy are affected and as such, they devalue themselves which demoralises them from forming meaningful relationships with others or working towards their life goals, all of which are important components for recovery.

“most of our patients with let’s say schizophrenia, have difficulty, or they feel that they, they will be stigmatised, I mean they also get very… they get lonely. So, they hope to get friends or even relationships, and I think they fear that they will be rejected.” [Psychiatrist]

“the effects of stigma are extremely damaging and counterproductive to that whole process because they know themselves without even people, you know, talking to them about their illness. They know themselves that they are in a manner disadvantaged, and that greatly reduces their self-esteem. That greatly reduces their self-esteem in being able to get to where they could have achieved, you know, even better. For example, finding someone that they like, getting a job that they like, improving other relations” [Pharmacist]

### Meso factors

The results also revealed that mental-health-related stigma is embedded in the community and social norms (meso level). The participants highlighted instances of dismissing, shaming, and excluding PMI and people associated with the stigmatised group (i.e., HP working closely with PMI).

#### Dismissiveness towards PMI

Public stigma may manifest in the form of people being dismissive towards PMI which could be discouraging towards their efforts at dealing with life’s challenges, as reflected in the verbatim listed below.

“when a patient comes in and says I am having difficulty finding a job, we always assume it’s their fault you know that? You know maybe, maybe you are…rather than saying “eh can I take a look at your resume?” or “what have you done? Where have you gone?” We immediately start to judge them.” [Counsellor]

“they have to put in extra effort to get school assignment done or do a normal day’s job compared to the average person who doesn’t. So, to them every day is a struggle, every day is a battle you know. And sometimes they didn’t get applause at the end of the day. All they get is all these mixed… sometimes wrong impression on the people that they work with. And I think that this part is not just in the workplace, in their personal relationships or in their studies, I find that it does affect…” [Pharmacist]

#### Associative stigma experienced by healthcare professionals

Two subordinate themes were identified pertaining to how stigma by association experienced by HP is a major contributor to feelings of burnout and compassion fatigue that subsequently hinders treatment and recovery of PMI.

##### Job devaluation

Participants revealed that often the value of their profession is minimised by the general public and peers. These concerns were particularly addressed by psychiatrists who disclosed that they were constantly compared to their peers (i.e., other doctors). Comparably, psychiatry is deemed a less reputable career choice. Individuals were also less encouraged to pursue psychiatry in medical school.

“I think that psychiatry, despite all the, you know, changes and the improvement is still stigmatised, so whenever you tell people you are a psychiatrist, people will laugh.” [Psychiatrist]

“come on la who wants to be a psychiatrist you tell me? Please, that’s not much money in it, you know. You know as a medical student, there is a stigma against psychiatry. Nobody ever encouraged us to take up psychiatry.” [General Practitioner]

##### Negative assumptions about HP

These responses illustrated the presumed notion that caring for PMI meant that one is more susceptible to mental illnesses, that mental illnesses are contagious, and that PMI are aggressive, therefore HP often have their safety questioned at work – such assumptions can contribute to feelings of frustrations at having to explain the misconceptions about their profession, further escalating job stress. While not directly affecting recovery, negative perceptions about their profession can spill over to the therapeutic work that HP conduct with PMI.

“A few of my friends in my previous line, not social line, they say “you also one day get infected in a way” you know, they say “maybe you go bonkers with them.” [Rehab Manager]

“At the same time, mainly the curiosity would turn to questions like “is your work very dangerous? How is the environment like? The patients there all...” Sometimes unfortunately they use the word “mad”. “So, a lot of mad people there, are they very aggressive?”” [Nurse]

Additionally, the public tends to erroneously view HP as self-sacrificial, altruistic figures that “take care” of these PMI. Whilst this can be seen as positive at face value, it has negative connotations attached to both the PMI and the role HP play in the diagnosis and treatment of PMI’s conditions.

“It’s interesting that you made a sacrifice to come into mental healthcare and what makes you want to make that sacrifice?” [General Practitioner]

“Whereas in mental health the general connotation with it ah, even though they may not say but I think, I think relates to like charitable work.” [Pharmacist]

### Macro factors

Participants highlighted that PMI experience discrimination and exclusion within healthcare providers and other systems such as employment and legal systems (macro-level). Three sub-themes were identified under macro-level factors, namely stigma within the healthcare settings, cultural norms within the Singapore society, and structural stigma.

#### Stigma within healthcare settings

Five subordinate themes were derived pertaining to how stigma within the healthcare settings results in suboptimal treatment for consumers. The former three themes encompass ways in which stigma is manifested, and the latter two themes encompass the reasons behind healthcare professionals’ stigma.

##### Diagnostic overshadowing

Diagnostic overshadowing is the tendency for HP to attribute consumers’ complaints or symptoms to their existing mental health conditions [53, 54]. Several participants provided accounts that alluded to the existence of diagnostic overshadowing in Singapore.

“In general healthcare, what some of my patients’ experience is when they sort of know that they have like depression, for example, they… okay again it goes both ways. So sometimes the healthcare practitioner in another hospital may see the condition and start to think “oh all of your somatic symptoms are… may be related to your depression” [Psychologist]

##### PMI being rejected by other H﻿P

Another implication of stigma within the healthcare system discussed by participants is the HP’s inclination to reject serving PMI, potentially delaying their access to proper treatment or giving patients suboptimal treatment.

“it might be just my perception or maybe my, you know… I’m not very clear whether it is because of stigma therefore they are making that kind of decision, that they don’t want my patient… to be in their hospital. You can send to other general hospital but don’t send to my … my hospital.” [Nurse]

##### Therapeutic pessimism

Participants were asked to opine on the extent of recovery possible for PMI, and only a minority of the participants gave responses that indicated that full recovery for service users is possible. Others had a less optimistic outlook on recovery, remarking that not everyone can recover and that recovery is contingent on the type of illness, indicating that therapeutic pessimism is prevalent in the healthcare system in Singapore.

“we have to accept that there are some conditions that are treatable and that whilst you’re on medication you are optimised and you are in remission. There are some conditions that we can treat and doesn’t come back, like anxiety like a phobia of some sort. There are some conditions that are chronic and even with the best of treatments you will still be having that condition with the symptoms and impacts on your life.” [Psychiatrist]

##### Peer and media influence on HP

When discussing the possible reasons for stigma among healthcare professionals, a common theme that was identified discussed the influence of peer and media on HP’s beliefs and attitudes towards PMI. PMI are often misrepresented in many media avenues, and as a result, professionals too are not immune to influence from this misinformation. Another avenue was through peer influence from fellow professionals in the field who have had encounters with PMI. When fellow peers share stories of negative experiences with PMI, healthcare professionals may experience anxiety that such an experience would occur to them which in turn results in them being fearful of PMI.

“You know when people don’t understand, they start to form certain preconceived notions or they form their own idea that eh, this is not ideal, and that has a… there are some consequences to it – colleagues or workmate right gather together they say, “hey there…” and it spread and you know it manifests and, so it does create some discrimination, and of course prejudice as well.” [Counsellor]

“because they have no encounter with a person with mental illness, then they read it in the newspaper. Or maybe they have some experience, I’m not sure, maybe they have some bad experience. So, when the person declares that they have mental illness, it gets their attention and they have to be… they are very wary, they will try to be careful. I think they just try to be careful.” [Nurse]

##### Inadequate training of HP

Participants also mentioned the lack of adequate training among healthcare professionals in mental health settings with regards to working with and providing services for PMI. Inadequate training of these professionals, especially new professionals in the field, affects their perceived and actual self-efficacy and knowledge. As such, these professionals tend to be anxious, fearful, and less confident when interacting with PMI, holding onto their preconceived negative notions about PMI.

“Maybe they are worried about their competency, knowledge, and skill in nursing this group of patients. Because you know our training as a student nurse… And during the 3 years, 2 years, they may not have a lot of encounters with people with mental illness. So, if they happen to be posted here or because we’ve sponsored them, so I think maybe it’s their competency is their fear whether “I can manage, I can nurse this group of patients.” [Nurse]

#### Local cultural and religious values

Three subthemes were derived that elucidated how local cultural level stigma determinants can influence PMI’s help-seeking behaviours and recovery.

##### Misattribution of illness due to cultural value

Several participants elaborated on how cultural values, more specifically Asian cultural values, tend to ascribe mental illness to the person’s weakness or inability to take hardships, undermine the severity of the illness and impede the help-seeking intentions of PMI.

“our own Asian culture, the earlier generations will always assume that you have to be hardy, you have to overcome problems. So, I think that’s one view that they will hold very closely, they will share with you, if you ever reveal to them that you have depression they will say “I also have what! But I cope with it what!” so it’s like you shouldn’t make a deal out of it.” [Psychologist]

One participant also expressed how the misattribution of the illness on the family’s part could be due to poor mental health literacy and can also lead to a delay in seeking appropriate help for the service user.

“I think the student does, but I think she was in a condition, a situation whereby she couldn’t…she needed help and she knew she needed help, so I think she identified that. But what wasn’t identified at that moment was her mum couldn’t see that as a mental health condition. She thought it was it just puberty yeah, so that to me was the discrepancy that was mentioned. So, it wasn’t so much the patient, but rather the family.” [Occupational Therapist]

##### Culturally influenced shame which impedes help-seeking

A few participants also discussed how cultural influences confers a notion of shame to mental illness which discourages help-seeking. This is especially pertinent in the Chinese culture, where an individual may feel discouraged to seek help to avoid bringing shame upon the family.

“particularly Chinese I guess, it’s something about a lot of shame with any kind of illness, regardless it’s mental or physical and more so with mental because I think they do believe that it’s something you can control. And it also makes it…there’s a Chinese saying, ‘don’t wash your dirty linen in public’ and that’s why therapy is strongly discouraged.” [Psychologist]

The same participant also cited how cultural ideologies of masculinity can impart shame on males wishing to seek help.

“gender is like the men have certain stereotypes they have to cope with? They feel more embarrassed about seeking help than to admit that they do have a mental condition.” [Psychologist]

On the contrary, individuals who are more infused with western cultures tend to be more indifferent towards the stigma associated with help-seeking, as highlighted by a participant, further accentuating the negative influence that Eastern culture has on help-seeking.

“the woman that seek help with us right are the ones that are insightful, so all these non- Singaporeans tend to come, by that I mean the westerners, they are the ones that self-select to see us, they are not the ones that have a stigma, feel the stigma of getting help.” [Psychiatrist]

##### Deferring appropriate help-seeking due to spiritual reasons

The attribution of mental illness to supernatural or religious etiological causes was also brought up by participants as a factor that results in the deferment of service users receiving appropriate professional mental health care.

“generally, Christianity. So, yea some of them say “oh you just need to pray” or “you didn’t pray hard enough” and so forth” [Psychologist]

“I think there is a group of people still among the religious establishments in Singapore, so for example the Ustaz and the Ustazahs (Islamic religious leaders), they may show a bit of a nonchalant attitude. Maybe these are those to me that think people with mental health issues need not see a doctor, really they should just seek spiritual help and improve their spirituality and then they will be alright.” [General Practitioner]

#### Structural stigma

Structural stigma is the last theme identified under macro factors. Participants mainly mentioned three structural level stigmas that compound the recovery for PMI.

##### Declaration of mental health history

The first has to do with the need to declare one’s mental health history during a job application. Having to declare one’s mental health history could result in unfair employment practices towards PMI, insomuch that they might be occluded from being hired if they do declare their conditions. And even if the said PMI does end up getting hired, they might be passed up for promotion or unfairly paid because of their illness.

“Now, even now, let’s say I want to send someone, to work. No high (er up), no employer will pay them that full rate, if they declare they have a mental illness.” [Medical Social Worker]

“employment? If they openly declared, they may have, certain conditions in there, to say that “ok, that person has a mental illness, so if you want to promote, do you think can cope a not?” [Psychologist]

##### Having a diagnosis precludes PMI from getting insurance

On a related note, a handful of participants mentioned that the need to declare one’s mental illness history usually precludes them from getting insurance coverage.

“it’s like “when are they ever going to convince that I can buy this? So, does that mean I can never be covered?” so…insurance is one big thing, it’s a very structured and systematic kind of like, they cannot buy, even for critical illnesses they cannot buy! So there’s certain things that’s like, why is that even a play, in matters like that?” [Psychologist]

##### Denying PMI equal opportunities

Additionally, structural stigma also hampers the client’s recovery as it denies them from having equal opportunities.

“We are talking about kids who have actually symptoms that are not so bad, but actually people are stigmatising, not giving them the opportunities, right? And that’s impacting them.” [Psychiatrist]

“so, the stigma means they definitely have less opportunities because there’s not, you know, we don’t have… you know many employers are still prejudiced against people with mental illness. So, there’s a limited pool of employers willing to give them a chance in the first place.” [General Practitioner]

##### Stigma associated with IMH

The last has to do with the stigma associated with IMH which could deter patients from seeking help at IMH, even though IMH is the only tertiary mental health hospital and arguably provides the most affordable psychiatric services in Singapore.

“Because they stigmatise themselves, actually the clients. Even when they go to IMH, they feel that it’s a mad hospital to them” [Rehabilitation Manager]

“a lot of times, you cannot even mention the word IMH at the beginning because people will like “ugh, why should I go IMH? I am not a mad man.” They will tell you. So, if you talk about that then that is…there is a lot of…I think a lot of times, there’s a lot of fear because of uncertainty. They, is not…is they don’t understand. To them IMH equivalent to? Mad. Right? So, they wouldn’t want to go.” [Medical Social Worker]

## Discussion

This study adopted a stigma perspective towards understanding recovery, and elucidated HP’s viewpoints on how the stigmatisation of mental illness affects the recovery for PMI, the findings of which were organised using a multilevel approach inspired by the earlier works of Logie et al., 2011 [41]. These themes were categorised into different levels to better conceptualize a model that elucidates mental health stigma in our findings (see Fig. 1). While the outcomes of this study may not be completely unique, given that certain features of stigma are ubiquitous, there are some salient points worth discussing.

Our analysis indicates that stigma on a personal level can have quite a deleterious effect on the individual’s willingness to seek help or even acknowledge their illness in the first place. A systematic review of 14 studies sheds light on some of the effective features of interventions that reduce self-stigma, such as empowering PMI and improving their self-esteem, both of which are key components of recovery-oriented practice [55, 56]. The findings of this study also observed the detrimental effect of cultural influences in Singapore on recovery. As elaborated by participants of this study, cultural influences associate mental illness with shame, which inhibits the help-seeking intentions of the consumer. This finding is consistent with a previous local study by Tan et al. [44], which showed that cultural misgivings towards mental illness such as the Chinese concept of “face” [57] contributes to the stigma of mental illness for it imparts shame to the sufferer and possibly their families as well [39]. Additionally, analogous to the findings of the present paper, a qualitative study exploring the viewpoints of Chinese medical students in China also highlighted the concept of “loss of face” that was deeply integrated in the society’s treatment of both PMI and individuals associated with it [58]. According to the study, this cultural factor is also reflected in the devaluation of the field of psychiatry, with Chinese medical students expressing that the field of psychiatry is often undervalued, and poorly taught and that psychiatric facilities are often underdeveloped [58]. Cultural influences may also compound the recovery process for PMI on an interpersonal level. Participants in this study mentioned the attribution of mental illness to personal weaknesses or supernatural causes (both of which are linked to culture) by the people close to the PMI, which may result in the PMI facing greater resistance to seeking professional psychological help. Consequently, there is a delay in receiving formal treatment, and studies have shown that a greater treatment gap is associated with adverse outcomes [59–61]. Such a finding lends credence to the fact that friends and families do influence an individual’s help-seeking intentions, thus reinforcing the importance of improving the mental health literacy of the population and addressing the cultural misgivings about mental illness in Singapore which leads to stigmatisation as discussed by Tan et al. [44].

Our findings suggest that stigma also permeates the healthcare settings in Singapore, as reported by our participants who had witnessed instances of diagnostic overshadowing by other HP. This corroborates the evidence from a previous study that documented the stigma experienced by PMI in Singapore, where PMI opined that their opinions were often disregarded by the HP [38]. In line with our findings, the literature suggests that diagnostic overshadowing is a global occurrence, which could delay consumers from receiving proper treatment and increase the risk of further health complications [53, 54]. Although there are other possible causes of diagnostic overshadowing, such as when HP are facing time pressures or when the workload is hectic, the accounts provided by our participants seemed to imply that the diagnostic overshadowing in this context is due to stigma, in the sense that HP were unable to see beyond the PMI’s diagnosis. Another healthcare-related stigma that participants elaborated on pertains to HP’s inclination to reject providing care to PMI because of a desire for greater social distancing. Therapeutic pessimism is another manifestation of healthcare stigma identified in this study, with many participants intimating that PMI will not be able to fully recover. This aligns with studies conducted overseas, suggesting that it is not an uncommon form of stigma [11, 62, 63].

As mentioned in the introduction, HPs may also contribute to the stigma towards mental illness, which in itself may present as a barrier to PMI’s recovery, and our study found that inadequacy in training was one of the key reasons for this stigma. Participants highlighted the issue of inadequate training in the mental health field, whereby many HP are thrust into the field without having adequate support. Some stated that when first joining the field, they had low perceived and actual self-efficacy, and a lack of knowledge with regards to PMI. In that case, HP at the nascent stage of their career are likely to be oblivious to their preconceived negative beliefs and attitudes towards mental illness. Moreover, they are also more susceptible to misinformation and misconceptions about mental illness from their peers and the media as compared to their more veteran peers as they are less likely to have developed the tools and first-hand experiences to counter misattributions. This was especially seen among professions whereby their training programmes do not allow for experiences with PMI – for instance, responses revealed that nursing programmes do not have mental health training before their posting to mental health services. The lack of proper training often leads to HP not being adequately prepared to interact with PMI or debunk negative attitudes and myths - these issues often lead to anxiousness and fear when interacting with PMI, resulting in a tendency to avoid PMI and desire for a greater social distance towards PMI [11]. Studies have also shown that healthcare professionals hold more negative attitudes towards their patients when they perceive that interacting with them is difficult, which often leads to PMI’s feelings of frustration and rejection [64–66]. As a result, this ultimately jeopardises the therapeutic relationship, adherence to treatment for PMI, and ultimately recovery [26].

The study also explored associative stigma experienced by HP from the perspective of hindering recovery among PMI. Salient issues highlighted were the negative assumptions the public have about HP, and a general pattern of job devaluation experienced by HP. Similar studies have addressed this issue, whereby HP working closely with PMI are often viewed as less skilled and less competent than their counterparts [19, 21, 67]. Halter [18] described it as nurses in the mental health field seen as “not real nurses”, a similar issue that was brought up by a psychiatrist in this study [19]. Furthermore, research encompassing medical students’ views has suggested that the general reputation of psychiatry is poor, citing reasons such as lesser respect and prestige compared to other specialities as focal reasons for opting to not specialise in psychiatry [68, 69]. This is akin to some of our participants suggesting that they were discouraged from joining psychiatry during medical school. A recent Singaporean study revealed that while doctors in mental health settings were more likely to experience moderate stigma, nurses were more likely to experience both moderate and high associative stigma [70]. Negative perceptions about these HP might spill over to their jobs and increase job stress [20, 71]. Previous studies have emphasised the connection between associative stigma and burnout, dissatisfaction, and compassion fatigue, which negatively affects the way practitioners interact with PMI, jeopardizing the quality of care [18]. Additionally, HP’s associative stigma has also been linked to higher levels of self-stigma among PMI [20], which as aforementioned, is detrimental to the recovery process.

Lastly, our findings also unearthed practices at a structural level that HP perceived as discriminatory, and exacerbating the challenges that PMI face in their recovery. The first would be the need for mental illness declaration on the job application forms, which is fortunately not as concerning presently because, at the time of writing, the Tripartite Alliance for Fair & Progressive Employment Practices in Singapore had already introduced a guideline to safeguard fair employment practices which proscribes companies from asking about mental health history on their job application forms unless it is a job-related requirement [72]. As mentioned by some of our participants, the need to declare one’s mental health history still has repercussions because purchasing insurance is still an area where PMI face structural discrimination for having a diagnosis. Anecdotal evidence suggests that having a psychiatric diagnosis often jeopardises one’s insurability even if their mental health condition does not implicate their physical health, and this could constitute as a barrier to help-seeking due to the aversion to being labelled with a psychiatric diagnosis [73–75].

Perhaps a difficulty that insurance companies face in providing PMI with coverage for physical health problems is that people with serious mental illness are associated with a higher risk of physical comorbidity and a shorter lifespan [76]. However, for insurance companies to exclude PMI who have sustained recovery and are otherwise physically healthy from getting insurance plans that provide physical coverage or to charge them higher premiums would be inequitable. It has been proposed by mental health advocates and stigma researchers for anti-stigma initiatives to not only concentrate on “soft goals” such as public education and changing attitudes but also to shift the focus towards addressing “hard goals,” in the form of legislative and policy change to promote social equity and improve the overall quality of life for PMI [77]. As such, to mitigate this particular barrier to help-seeking, there may be a need for policymakers to press for legislative changes in the realm of insurance. For instance, insurance companies could shift towards a case-by-case basis to evaluate applications from PMI and be more transparent about their underwriting process, instead of rejecting PMI without providing concrete reasons. Such a shift could reduce stigma on a structural level and promote equity for PMI, concomitantly sending across a message that recovery is possible and that PMI are not markedly different from the lay public.

An interesting finding that our study came across is the stigma associated with IMH (the state mental hospital) which could potentially deter individuals from seeking help, or more specifically seeking help from IMH. An earlier local study showed similar results, where it was reported that for individuals with non-schizophrenia disorder, greater stigma was associated with being treated by IMH as compared to being treated in a university hospital [78]. In contrast, individuals with schizophrenia in that study reported a greater degree of stigmatisation in general hospitals as compared to at IMH. Chee and colleagues [78] posited that their finding might be attributable to the fact that there are disease-specific and institution-specific aspects of stigma because the proportion of patients with schizophrenia only comprises a fairly small percentage in the general hospital and so the demographic of psychiatric patients in the general hospital is more heterogeneous, whereas IMH comprises largely patients with schizophrenia as it caters to most of the persons with schizophrenia in the country. In our study, participants implied that many individuals are aversive towards IMH for they equate it to a “mad hospital”, and terms such as psychotic and madness are typical stereotypes associated with schizophrenia [79, 80]. By juxtaposing the explanation by Chee and colleagues with this study’s findings, it can be postulated that the stigma towards IMH could very likely be because of an intertwining of disease-specific (schizophrenia) and institution-specific stigma. Another plausibility for this aversion towards IMH could be the fact that a general hospital treats a variety of health conditions, and one could better conceal their mental health condition by seeking treatment at a general hospital [81, 82]. Nonetheless, it is recommended that more studies be carried out to affirm the hypotheses of this finding, as well as research for effective strategies to eradicate such stigma.

There is a growing body of research in Canada documenting the effective strategies and key ingredients for the reduction of stigma in healthcare settings [11, 83], which includes educating HP on “what to say” and “what to do”. To attenuate the impact of healthcare stigma identified in this study, there is a need to also focus anti-stigma efforts on HP, and we postulate that it would be helpful to take reference from the work by Knaak and colleagues [84]. For instance, teaching HP in the general healthcare system about “what to say” and “what to do” would arguably reduce HP’s feelings of inadequacy in working with PMI. Knaak et al. also advocated for social contact approaches with HP in a non-typical provider-patient interaction, such as having PMI share about their lived experience of illness and recovery as well as their experiences in healthcare settings. Such forms of social contact are likely to increase empathy and diminish fear of PMI. Conceivably, the reduction of stigma would also lead to a decrease in instances of diagnostic overshadowing or the tendency for HP to reject working with PMI.

Knaak and colleagues also proposed emphasizing that recovery is possible and demonstrating the impactful roles that HP play in this process [11, 84], which could alleviate some of the pessimistic views about recovery held by HP. Implementing recovery-oriented models of care would probably be another effective approach to counteract therapeutic pessimism. This way, recovery would no longer be framed as an end state characterised by the decrease of symptoms and disabilities. Rather, when recovery is regarded as a process as in recovery-oriented practice, the aim would be to support PMI in a way to inspire hope and see beyond the illness, as well as giving more agency to the PMI in their recovery goals setting [4, 55]. Moreover, some studies have shown that recovery-oriented practice is associated with better therapeutic alliance [85, 86], and research has indeed evinced that better therapeutic alliance is linked to positive outcomes such as reduction of psychiatric symptoms and improvement in quality of life [87], further substantiating the advantage of implementing recovery-oriented practice.

Additionally, the vulnerability of HP working closely with PMI to associative stigma calls for implementations that address the challenges that healthcare professionals might experience. It is important that healthcare professionals, especially those new to the field, are aware of such stigma, and able to identify how it affects their job-related tasks. Bladon suggested that one possible way of mitigating stigma is by celebrating the uniqueness of these professionals through public means [88]. Emphasising the unique and positive contributions of HP working with PMI through public education does not only increase the positive identity of these professionals but also possibly can enhance client outcomes through reducing mental illness-related stigma and giving a platform for HP to copiously advocate for patient care [89]. Even though associative stigma is experienced, it is essential that these HP still can maintain pride in the profession and acknowledge that the work that they do is valuable [21].

### Limitations

There are a couple of limitations pertaining to this study that need to be highlighted. Firstly, most of the participants in this study were affiliated with IMH, and it is possible that the findings of this study would not be generalizable to HP working in other hospitals or private settings. Secondly, the sample of this study consisted of HP from various occupations, and there might be unique viewpoints from the various specific occupations which were not elucidated in this study. Furthermore, since participation was voluntary, it is probable that many of our participants are strong advocates of anti-stigma work, and they may hold views that are disparate from those who are not. Lastly, although participants were assured of confidentiality, it is possible that they were not completely candid in their discussions and had withheld some personal views, which could be in part attributed to social desirability bias as well as a fear of expressing opinions that might have implicated other organizations or hospitals. Based on the above limitations, it is recommended for future studies to sample only HP of a particular occupation, or to include only HP from general hospitals and private settings, to allow for a more diverse understanding of how stigma influences recovery. These limitations notwithstanding, our study presents an early attempt to examine how stigma influences recovery from the perspective of HP, and also showcased important insights on the challenges that stigma poses toward recovery, the findings of which could inform policymakers of ways to improve the recovery of PMI.

## Conclusions

The results of this study serve as a buttress to Angermeyer and Schomerus’s [9] argument that the stigma perspective could eradicate some of the blind spots of recovery. The present study elucidated several determinants of stigma from the perspective of healthcare professionals working closely with PMI. A total of 17 themes were derived, and these were classified into a socioecological model to demonstrate stigma across micro, meso, and macro levels. Findings from the study illustrated that some of the viewpoints articulated by healthcare professionals have been pervasive among other stakeholder groups – internalised stigma (micro) and cultural factors (macro) are factors that the general public have also illustrated as prevailing determinants of stigma [39]. Perhaps more unique to our study is the stigmatisation within the healthcare system and healthcare professionals, as both stigmatisers and stigmatised, and its impact on PMI’s therapeutic process and recovery. Lastly, based on our findings, we have proposed the implementation of some “soft goals” and “hard goals” in our fight against eradicating stigma to support the recovery of PMI in Singapore.

## Supplementary Information

**Additional file 1.**

## Data Availability

The authors’ government law and institution only permits sharing of human participant data with researchers with whom they have a written agreement. These restrictions have been imposed by our Institutional Review Board (IRB) and Institutional Committee (NHG Domain Specific Review Board and IMH Clinical Research Committee). Our IRB guidelines suggest that a Research Collaboration Agreement (RCA) be signed with collaborating parties. However, data sharing with clear research purposes are available upon request at this contact: Research Director of IMH, Associate Professor Mythily Subramaniam (mythily@imh.com.sg).
