# Supplementary material for: The perspectives of healthcare professionals in mental health settings on stigma and recovery - A qualitative inquiry
Source: BMC Health Serv Res. 2022 Jul 9;22:888. doi: 10.1186/s12913-022-08248-z (PMC9270770; doi:10.1186/s12913-022-08248-z)
Supplement: Supplementary file 1 — Additional file 1. [file 12913_2022_8248_MOESM1_ESM.docx]

**Preliminary SSI Guide for Care Providers**

- Can you tell us in your own words what does ‘stigma’ mean to you?
- Can you give us examples of stigmatizing behaviour?
- Do you think people in general would have any negative perceptions towards persons with mental illness? Can you describe some of these negative perceptions they might have?
- What do you think could be some of the causes for stigma towards people with mental illness to arise in Singapore? If needed
- Why do you think some people may hold stigmatizing views towards mental illness?
- Some people believe that culture plays a role in stigma. What are your thoughts? If so how does it impact stigma?
- Have your patients ever talked to you about experiencing unjust, prejudicial, or discriminatory (stigmatizing) behaviour because of their mental illness? Can you tell us more about it?
- How do you think this affects those with mental illness?
- Have your patients sought your advice on revealing their mental illness to family members or employers? What advice do you usually give them?
- Do you think that other healthcare providers hold negative views towards those with mental illness? Can you tell us more about it?
- Have you ever felt that people in general or even professional colleagues have any negative perceptions towards you given your current role in working with those with mental illness?
- Can you describe what you think are some of the ways/strategies that can be used to reduce stigma towards people with mental illness in Singapore?
- Are you aware of the term structural stigma? What does this term mean to you?

We have reached the end of the interview. Is there anything more you’d like to tell me that we have not already discussed?

Do you have any questions for me about anything we have discussed?

**Summarise the SSI for participant.**
